# Recent Advance on Biological Activity and Toxicity of Arecoline in Edible Areca (Betel) Nut: A Review

**DOI:** 10.3390/foods13233825

**Published:** 2024-11-27

**Authors:** Gang Huang, Deyong Zeng, Tisong Liang, Yaping Liu, Fang Cui, Haitian Zhao, Weihong Lu

**Affiliations:** 1 School of Chemical Engineering and Chemistry, Harbin Institute of Technology, Harbin 150001, China; zhongao8888@126.com (G.H.); cuifang@hit.edu.cn (F.C.); 2 Chongqing Research Institute of Harbin Institute of Technology, Chongqing 401151, China; zengdy@hit.edu.cn (D.Z.); liangts2023@126.com (T.L.); 17320064038@163.com (Y.L.); 3 School of Medicine and Health Sciences, Harbin Institute of Technology, Harbin 150001, China

**Keywords:** arecoline, toxic effects, biological activity, organ systems

## Abstract

Areca nut (*Areca catechu* L. AN), which is the dried, mature seed of the palm species *Areca catechu* L., is consumed by over 600 million individuals, predominantly in South Asia, East Africa, and certain regions of the tropical Pacific. The International Agency for Research on Cancer (IARC) has classified it as a species carcinogenic to humans and designated it as a Group 1 human carcinogen. Arecoline, which has attracted attention for its therapeutic potential in the treatment of mental illness and the relief of gastrointestinal disorders, is the main active alkaloid in the areca nut. However, in 2020, the IARC said that arecoline might be a “probable human carcinogen”. Arecoline can cause various types of cellular damage, primarily leading to the destruction of cell morphology, reduced survival rates, abnormal physiological functions, and even cell apoptosis. The research on its toxic mechanisms includes several aspects, such as increased levels of reactive oxygen species, autophagy, epigenetic dysregulation, and immune dysfunction, but these research findings are scattered and lack systematic integration. This article summarizes the effect mechanisms of arecoline on the oral cavity, neurological and cardiovascular systems, and other organs, as well as embryogenesis, and provides detailed and valuable insights for the clinical practice and targeted therapy of arecoline.

## 1. Introduction

The areca (betel) nut, derived from the seed of the areca nut palm, ranks as the world’s most prevalent harmful and addictive substance, following tobacco, ethanol, and caffeine [1]. In 1992, the World Health Organization (WHO) recognized the areca nut and tobacco as direct causative agents of oral squamous cell carcinoma (OSCC). Subsequently, in 2003, the WHO classified the areca nut as a primary carcinogen [2]. To date, more than an estimated 600 million individuals are habitual users of chewing the areca nut, with most in Central and South Asia, Southeast Asia, and Melanesia, including India, Pakistan, Bangladesh, Myanmar, Malaysia, Taiwan, and other countries [1,3]. The pharmacological and toxicological effects of the areca nut are dose-dependent. At low doses, it exhibits repellent and anti-inflammatory properties, enhances gastrointestinal function, modulates blood lipid levels, and offers protection against atherosclerosis. Additionally, it has been noted to have antidepressant effects. However, prolonged consumption of the areca nut can lead to adverse reactions in the human body and may be associated with malignant diseases, such as cirrhosis and oral submucosal fibrosis, among other toxicological effects [3,4].

Arecoline (N-methyl-1,2,5, 6-tetrahydropyridine-3-carboxylate methyl ester), the primary alkaloid found in areca, has garnered significant interest within the realms of medicine and biology due to its pharmacological properties and toxic effects. Recent advancements in research pertaining to the physiological functions of areca alkaloids have led scholars to increasingly recognize their detrimental effects on human health. These encompass cellular damage to vital organs, such as the brain, heart, lungs, digestive tract, and genital system, as well as alterations in cell morphology. Such damage can precipitate conditions like oral submucosal fibrosis and various forms of cancer [5]. Furthermore, prolonged consumption of the areca nut may result in damage to both the reproductive and urinary systems. This could potentially impair sperm count and activity, as well as affect the acrosomal reaction of sperm and its ability to fertilize [5]. It significantly impacts the functionality of the nervous system, suppresses its cellular antioxidant capacity, results in oxidative stress damage, and elevates the prevalence of nervous system disorders [6,7]. However, some studies have shown that low doses of arecoline in the areca nut have therapeutic effects for some diseases [4], such as its role in bone disease [8], Parkinson’s disease models [9], and Alzheimer’s disease [10,11,12]. To comprehensively understand the multifaceted nature of areca alkaloids, numerous studies have been conducted. These investigations encompass quality improvement techniques and extraction and analysis methodologies, as well as the metabolism of areca alkaloids in various environments. These studies offer valuable insights into the areca nut and its alkaloid constituents, suggesting potential avenues for the formulation of novel therapeutic approaches.

In this work, we searched the following databases using arecoline, toxicity, and biological activity as the keywords: The Web of Science, Science Direct, and PubMed. Three authors independently screened the titles and abstracts of all relevant papers in recent years, followed by full-text screening. Any conflicts were resolved by the three authors through discussion and consultation with a fourth author. Finally, 95 articles were reviewed, and the research content was summarized, focusing on the types, detection methods, pharmacological effects of arecoline, and its effects on different human organs, aiming to review and prospect for the related fields of arecoline and provide references for subsequent studies.

## 2. Species and Identification Method of Areca Alkaloids

**Figure 1:**
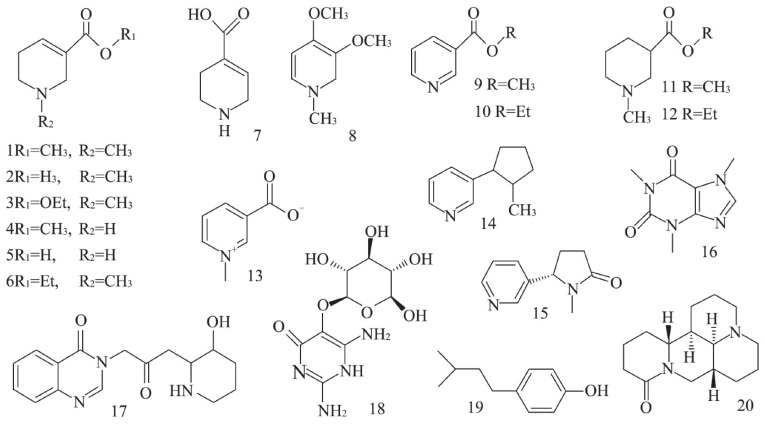
The alkaloid structures in several common areca nuts [16]. (1. arecoline; 2. arecaidine; 3. arecolidine; 4. norarecoline hydrochloride; 5. guvacine hydrochloride; 6. N-methyl-1,2,5,6-tetrahydrogen-pyridine-3-ethyl carboxylate; 7. isoguvacine; 8. homoarecolin; 9. methyl nicotinate; 10. ethyl nicotinate; 11. N-methylpiperidine-3-methylcarboxylate; 12. N-methylpiperidine-3-ethyl carboxylate; 13. trigonelline; 14. nicotine; 15. cotinine; 16. caffeine; 17. febrifugine; 18. vicine; 19. hordenine; 20. dophoridine).

The alkaloids in the areca nut are mainly pyridine alkaloids, and the total alkaloids are about 0.3~0.7%. It has been reported that about 20 alkaloids (Figure 1) are found in the areca nut, among which arecoline, arecaidine, guvacoline, and guvacine are the most common [12,13,14,15,16]. The chemical structure of areca alkaloids has double bonds at the exact location of the ring and contains ester bonds and nitromethyl functional groups, and its chemical structure is shown in Figure 1 [16]. Arecoline and norarecoline are hydrolyzed products of arecaidine and norecaidine. The separation, purification, and determination methods of these four alkaloids have been studied and explored. The extraction methods of areca alkaloids mainly include solvent extraction, ultrasonic-assisted extraction, subcritical water extraction, and supercritical CO_2_ extraction [17,18]. Currently, the primary detection methods for arecoline that have reached a significant level of maturity encompass high-performance liquid chromatography, ultra-high performance liquid chromatography–tandem mass spectrometry, microchip capillary electrophoresis with contactless conductance detection, and gas chromatography–mass spectrometry [17]. Dai et al. [19] employed near-infrared spectroscopy (NIRS) to quantify the primary active components of the areca nut, namely, gallic acid, tannins, catechin, and arecoline. The quantification of these four active components was further enhanced using high-performance liquid chromatography (HPLC). By integrating these findings, a rapid near-infrared detection model for the main active substances in the areca nut was established. Pan et al. [20] devised a method utilizing ultra-high performance liquid chromatography–MS/MS (UPLC-M S/MS) to simultaneously quantify areca alkaloids in rat plasma, following oral administration of the areca extract. This method also facilitated pharmacokinetic studies that indirectly elucidated the metabolic relationship of the four areca alkaloids in vivo. Furthermore, Pan [21] introduced a rapid, selective, and accurate high-performance liquid chromatography–electrospray ionization tandem mass spectrometry (LC-ESI-MS/MS) technique for the concurrent quantification of arecoline, arecaidine, and arecoline n-oxide in rat plasma. This comprehensive approach effectively validated their pharmacokinetic post-oral arecoline administration.

## 3. Toxicological Effects of Areca Nut

Arecoline is a key medicinal component of the areca nut and is soluble in water, ethanol, ether, and chloroform [21]. Extensive studies have linked it to the onset of various diseases. The ingestion of the areca nut has been associated with an elevated risk of developing oral squamous cell carcinoma (OSCC), oral submucosal fibrosis (OSF), and head and neck cancer. Furthermore, it is implicated in DNA damage and a range of systemic diseases, including atherosclerosis and cardiovascular disease [22]. Modern toxicological studies have reported that arecoline has oral submucosal fibrogenic toxicity, reproductive toxicity, hepatorenal toxicity, immunosuppressive toxicity, and neurotoxicity, and it has been observed to induce cell cycle arrest, apoptosis, and cytotoxicity in human oral endothelial cells [23], as well as having cytotoxic and genotoxic effects in hepatocytes [24], bone marrow cells (BMCs) [25], lymphocytes (PBLs) [26], and neuronal cells [27]. Moreover, arecoline has been implicated in neurodegenerative diseases such as Parkinson’s disease [9].

In recent years, many researchers have conducted in-depth studies on the pharmacological effects of areca alkaloids. The effects of areca alkaloids on various organs are shown in Table 1. It can be seen from the table that arecoline affects the oral system, the brain nervous system, the cardiovascular system, the gastrointestinal system, the liver system, and even embryos to varying degrees, and different doses of arecoline have different effects on each organ.

## 4. Effects on Inducing Oral Fibrosis and Oral Diseases

**Figure 2:**
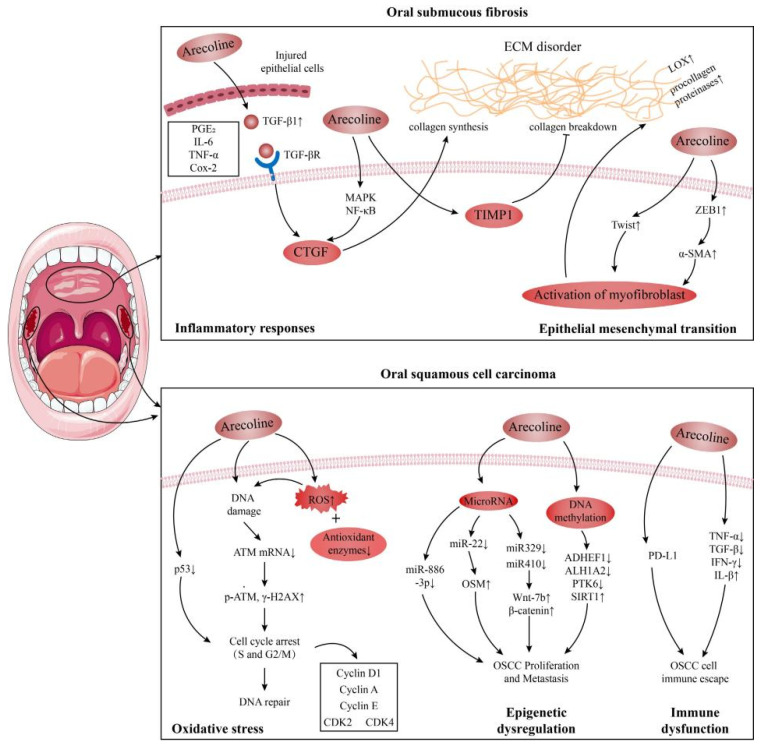
A potential mechanism for the development of arecoline-induced OSF and OSCC [45].

The International Agency for Research on Cancer (IARC) has designated the areca nut and its principal constituents as Group 1 carcinogens [41]. Oral cancer ranks as the seventh most prevalent cause of cancer-related mortality globally, with an estimated 710,000 cases annually. Dishearteningly, only 40–50% of those diagnosed survive beyond five years [42]. The elevated incidence of oral cancer is intimately associated with habits such as areca nut chewing, smoking, and alcohol consumption. Arecoline, a compound found in the areca nut, triggers the production of reactive oxygen species (ROS) and contributes to DNA damage during oral cancer progression [43]. Oxidative stress plays a pivotal role in this malignancy, primarily affecting DNA and proteins in normal cells, leading to carcinogenic mutations and the malignant transformation of the oropharyngeal mucosa and enhancing the progression and invasiveness of OSCC cells [44]. Both areca extract (ANE) and areca alkaloid exhibit genotoxic properties, promoting increased levels of ROS and resulting in chromosomal aberrations in mammalian cells [1]. Figure 2 presents potential mechanisms through which arecoline contributes to oral submucosal fibrosis (OSF) and oral squamous cell carcinoma (OSCC) [45].

### 4.1. Oral Submucosal Fibrosis (OSF)

Oral submucosal fibrosis (OSF) is a chronic oral mucosal disease characterized by chronic inflammation processes and persistent fibrosis proliferation. It represents a precancerous lesion that is causally associated with the consumption of the areca nut. As indicated by epidemiological studies, the primary etiological factor associated with OSF is chewing the areca nut [46]. Consumption of the areca nut has been demonstrated to exert a detrimental impact on the oral mucosa, precipitating inflammatory responses and fibrosis. This is attributed to the impairment of T-cell activation and the subsequent stimulation of the production of transforming growth factor β (TGF-β1), prostaglandin E2 (PGE2), transforming growth factor-α (TNF-α), and interleukin-6 (IL-6), which are pivotal in the pathogenesis of both cancer and OSF [47,48].

It has been demonstrated that the primary pathway associated with OSF is linked to a number of inflammatory mediators. These include but are not limited to transforming growth factor-beta (TGF-β) [49], PGE2, IL-6, TNF-α, and cyclooxygenase 2 (Cox-2). TGF-β is a pivotal mediator in the pathogenesis of OSF [48]. Areca-derived substances, such as arecaolin, induce keratinocytes to secrete a considerable amount of TGF-β by upregulating the TGF-β signaling pathway, which further affects the transformation of oral mucosal fibroblasts into myofibroblasts and deepens the role of fibrosis. Currently, the TGF-β pathway is understood to play a role in inhibiting collagen degradation and activating the inhibitor of the plasma plasminogen activator in the process of fibrotic diseases, affecting the balance between matrix metalloproteinases and inhibitors at DNA and protein levels, promoting the mesenchymal transformation of epithelial cells and resulting in the imbalance of collagen metabolism. Nevertheless, the induction of arecoline has been observed to elicit an overt oxidative stress response in fibroblasts, culminating in DNA damage, lipid peroxidation, and cellular death [50,51,52], thus promoting the induction of oral cancer. Concurrently, research has demonstrated that aprocoline can modulate phosphoinositide 3-kinase/protein kinase B (PI3K-Akt) and mitogen-activated protein kinase (MAPK) pathways. This modulation promotes the expression of inflammatory mediators, such as IL-6 and interleukin 8 (IL-8), induces collagen hyperplasia, and contributes to the development of oral submucosal fibrosis [52,53]. In the phosphatidylinositol 3-kinase-Akt pathway, the expression of protein kinase B (AKT1) is increased by activating the AKT1 protein downstream, which intensifies the expression and accumulation of the phosphorylated AKT (p-AKT) protein [54]. In the MAPK pathway, by upregulating the c-Jun N-terminal kinase (JNK) protein, the activity of the downstream AP-1 complex protein c-jun and c-fos transcription factors is induced to increase and promote their aggregation to the nucleus.

Another pathway of OSF is arecaine’s induction of epithelial–mesenchymal transformation (EMT) in human buccal mucosal fibroblasts (BMFs), which is a key factor in promoting tumor progression and fibrosis [55]. In the context of carcinogenesis and metastasis, the expression of specific proteins and enzymes is enhanced, leading to epithelial–mesenchymal transformation (EMT) and the subsequent activation of EMT-associated proteins. This process can be influenced by a variety of chemical compounds, such as ROS, TGF-β, Notch receptor-1, and inflammatory cytokines [56]. Zinc Finger E-box Binding Homeobox 1 (ZEB1) functions as a transcription factor, regulating its target genes via its protein-binding domain, with a particular emphasis on e-cadherin. The alkaloid arecoline has been demonstrated to induce the formation of α-smooth muscle actin (α-SMA)-positive stress fibers in cells that express the transcription factor ZEB1 in a manner that is dependent on the presence of the BMF protein. Furthermore, arecoline has been demonstrated to induce collagen contraction in the BMF. Chromatin immunoprecipitation demonstrated that arecoline enhanced the binding of ZEB1 to the α-SMA promoter in the BMF, thereby inducing the BMF to undergo transdifferentiation into myofibrocytes. Subsequently, it promotes extracellular matrix (ECM) accumulation and participates in the pathogenesis of OSF [57,58].

### 4.2. Oral Squamous Cell Carcinoma (OSCC)

Oral squamous cell carcinoma (OSCC) is a malignant tumor that can occur in various parts of the mouth, including the lips, tongue, salivary glands, gums, floor of the mouth, oropharynx, and buccal mucosa. Oral cancer ranks as the seventh most prevalent cause of cancer-related deaths globally, with an annual estimation of 378,500 new cases. Notably, half of the oral cancer-related fatalities occur in Southeast Asia [59]. OSF is a precancerous lesion of OSCC and can precede the diagnosis of OSCC [60]. Statistics show that the incidence of OSCC accounts for about 90% of oral malignancies. According to the Global Cancer Observatory (GCO), the incidence of OSCC will increase by about 40% by 2040, and the mortality rate will gradually increase [61]. Epidemiological research has demonstrated a strong correlation between the act of chewing the areca nut and the incidence of OSCC. The areca line found in the areca nut exhibits mutagenic and carcinogenic properties, thereby increasing the likelihood of malignant tumor development in patients with oral submucous fibrosis (OSMF) [62]. Concurrently, research has indicated that arecoline may contribute to the development of OSCC by inducing heightened oxidative stress, epigenetic irregularities, and immune system dysfunction [28,63,64].

Oral cancer is caused by the accumulation of oxidative stress in normal cells, resulting in damage to DNA and proteins. This leads to the development of carcinogenic mutations and malignant transformation of the oropharyngeal mucosa. Consequently, the progression and invasiveness of OSCC cells are enhanced. It has been demonstrated that oxidative stress can promote the occurrence, progression, and resistance to therapy of tumors by damaging the DNA of cells. This leads to the accumulation of mutations and genomic instability and results in the reprogramming of cell metabolism and signaling pathways [65]. The administration of arecoline has been demonstrated to induce an increase in ROS production and a reduction in the expression of antioxidant enzymes, which, in turn, results in DNA damage and the inhibition of DNA repair activity. This ultimately leads to the accumulation of error-prone DNA replication and mutation [66,67]. The administration of 100–200 μg/mL of arecoline resulted in DNA damage, the induction of phosphorylated ATM (p-ATM) and phosphorylated ATR (p-ATR) expression, and an increase in genotoxicity. Arecoline oxidative metabolites (ANOs) have been demonstrated to exhibit high levels of cytotoxicity, DNA damage, and proliferation activity in the initial stages of chewing arecoline. Furthermore, they have been shown to stimulate extracellular signal-regulated kinase (ERK), JNK, p38, and pro-inflammatory cytokines through peroxide stress and induce an increase in ROS, thus increasing the risk of OSCC [68].

## 5. Effects on the Nervous System

The nervous system is a functional collection of cells, tissues, and organs that enables the body to react to both internal and external stimuli in a controlled manner. The human nervous system can be broadly categorized into two principal sections: the central nervous system (CNS), which encompasses both the brain and the spinal cord, and the peripheral nervous system, which is constituted by the cranial and spinal nerves. The primary role of the human nervous system is to act as a central control, coordinating and regulating the functions of various organs and systems within the body. This ensures that the human body operates as a cohesive unit. The system controls movement, regulates the endocrine system, maintains cardiovascular stability, manages respiration, oversees the digestive system, and regulates the immune system, among other functions [69,70,71].

Consumption of the areca nut has been observed to produce a range of effects on the central nervous system. These include the induction of euphoria, palpitations, and salivation, as well as an increase in perspiration. Additionally, it has been noted to improve cognitive impairment and reduce anxiety. The nut also carries addictive properties and can suppress hunger while simultaneously enhancing physical activity [72]. Arecoline, the main alkaloid in arecoline, easily crosses the blood–brain barrier (BBB) [9] and stimulates nicotinic and muscarinic acetylcholine receptors, thus stimulating the central nervous system. The specific mechanisms of influence on the nervous system include neuron damage and nerve signal transmission. Arecoline induces neurotoxicity via oxidative stress-mediated apoptotic signaling. This process elevates reactive ROS levels, diminishes antioxidant concentrations, and impedes the protective mechanisms against ROS, thereby leading to neuronal damage [27]. Arecoline concentrations, ranging from 50 to 200 μM, have been observed to disrupt neuronal redox homeostasis, subsequently inducing neuronal cell death [27]. It was observed that the administration of arecoline resulted in the disruption of the autophagy flux in PC12 cells, which manifested as an increase in the number of autophagy vacuoles. Additionally, the levels of LC3II/LC3I and p62 expression were elevated in PC12 cells following the administration of arecoline. These changes led to a notable decrease in the viability of PC12 cells, thereby indicating a neurotoxic effect of arecoline on these cells [73]. This neurotoxicity is associated with endoplasmic reticulum (ER) stress and interference with endogenous H2E production [74]. Arecoline is also commonly used as an important central nervous system drug. Serikuly et al. [31] discovered through their experiments on zebrafish that a concentration of 10 mg/L of arecoline significantly upregulates the brain expression of two primary early proto-oncogenes, namely, c-fos and c-jun. These genes serve as biomarkers for neuronal activation. Moreover, this specific concentration of arecoline elicits distinct neurochemical effects. These include the activation of central monoaminergic neurotransmission, characterized by elevated levels of norepinephrine serotonin and 3,4-dihydroxyphenylacetic acid (DOPAC), as well as a reduction in the 5-HIAA/serotonin ratio, evidenced by decreased metabolite to serotonin or dopamine ratios. These findings suggest that arecoline has anti-anxiety characteristics, accompanied by monoaminergic neurotransmission changes [31]. Wang et al.’s [7] research also showed that norarecaline has significant anti-anxiety activity, and its mechanism of action is related to combating oxidative stress damage, inhibiting neuroinflammatory response, and regulating neurotransmitter levels and the N-methyl-D-aspartate receptor/calcium–calmodulin-dependent protein kinase II/protein kinase B (NMDAR/CamkII/Akt) signaling pathway. In terms of improving memory, studies have shown that low-dose arecoline can significantly increase the discrimination index (DI) of novel object recognition (NOR) and object location recognition (OLR) in mice, indicating that it can enhance physiological memory and improve memory disorders in mice [75]. Arecoline has been demonstrated to alleviate a range of neurological disorders, including depression, alcohol intoxication, schizophrenia, and Alzheimer’s dementia [12].

The aforementioned studies demonstrate the multifaceted impact of arecoline on the central nervous system. Elevated doses of arecoline can precipitate neurotoxicity, apoptosis, and carcinogenic transformation within the central nervous system. Prolonged ingestion and misuse of arecoline can result in addiction, tolerance, and dependence, facilitated by the dopamine release in the brain. Conversely, at lower concentrations, arecoline enhances the expression of neuronal biomarkers, thereby eliciting functions such as anti-anxiety effects and cognitive enhancement. Research into the beneficial effects of arecoline could offer novel therapeutic strategies for related diseases and hold potential significance for the development of new pharmaceuticals and future clinical applications.

## 6. Effects on Cardiovascular System

Habitual chewing of the areca nut will increase the risk of cardiovascular disease death, causing arrhythmia, sinus tachycardia, and arrhythmia [76], and can lead to cardiomyocyte apoptosis and myocardial infarction [35]. It can change heart function by inducing heart hypertrophy after cardiomyocyte apoptosis, causing heart injury [35]. Arecoline is considered a possible contributing factor to coronary spasms because of its sympathomimetic effect on the vascular endothelium [29]. Moreover, long-term chewing of the areca nut can lead to increased morbidity and mortality of cardiovascular diseases. Research has demonstrated that arecoline can not only induce cardiomyocyte apoptosis [35] but also activate several hypertrophy-related signaling pathways. These include the IL-6-induced MEK5/ERK5, JAK2/STAT3, mitogen-activated protein kinase, and calcineurin signaling pathways and can induce several hypertrophy-related signaling pathways. It causes cardiac hypertrophy after apoptosis of cardiomyocytes to change heart function and cause heart damage [77], and aprodine can promote protein expression of mitochondria-dependent and Fas-dependent apoptosis, inducing cardiotoxicity and apoptosis by inducing death receptors and mitochondria-dependent apoptosis pathways in the heart [35]. Ku et al. [34] administered arecoline via intra-peritoneal injection to male Wistar rats at either a low dose (5 mg/kg/day) or a high dose (50 mg/kg/day) for a duration of three weeks. The findings of the study indicated that arecoline has the potential to induce cardiac fibrosis through the accumulation of collagen. However, some researchers suggest that 5 mg/(kg.d) of arecoline can protect against myocardial damage caused by acute alcoholism [78].

## 7. Effects on the Reproductive Organs

The deleterious effects of arecoline on the human body extend beyond oral, neurological, and cardiovascular toxicity, also posing significant risks to other organs. Despite its addictive properties, some pregnant women persist in consuming the areca nut during gestation. In recent years, there has been a growing interest in the reproductive developmental toxicity of arecoline. Studies have revealed its impact on fertility and early embryonic development, demonstrating embryotoxicity in various species, including birds, mice, and zebrafish [79]. Several studies have investigated the impact of arecoline on mouse embryo development. The findings suggest that arecoline not only decreases the number of viable pregnancies but also inhibits the in vitro growth of blastocysts. This confirms that arecoline exhibits both embryological and blastocyst toxicity [39]. Yuan et al. [80] investigated the impact of arecoline on human sperm survival rates, employing three distinct concentrations of alkaloids to evaluate their motility (Sperm Motility, SM). It was found that arecoline has a lethal effect on human sperm, and this effect is dose-dependent. The effect of arecaidine is much weaker than that of arecoline, and the effect of demethylated arecaidine is the weakest. These results suggest that the habitual chewing of the areca nut is detrimental to human gonadal function [80]. Er et al. [81] investigated the impact of arecoline on human sperm survival and cyclooxygenase-2 expression. The study utilized three distinct concentrations (50, 100, and 200 mg/L) on male semen. The findings revealed that arecoline can diminish both the motility and survival rate of sperm. The underlying mechanism may be associated with arecoline’s induction of sperm cell cycloxygenase (cyclooxygenase-2, COX-2) expression. This is because the genes of COX-2 are not inherently expressed and must be stimulated and induced by certain cytokines, pro-cancer factors, NO, or proinflammatory factors. The use of arecoline to induce the gene expression of COX-2 leads to a sustained weakening of sperm motility. This conclusively demonstrates that arecoline is detrimental to human reproduction [81]. The study of Zhou et al. [82] demonstrated that arecoline targets the male reproductive system, inducing a multifaceted response. This includes an increase in reactive oxygen species (ROS), leading to oxidative stress and an upregulation of tumor necrosis factor α levels. Additionally, it induces the overexpression of COX-2, which impacts the immune system and subsequently damages sperm. Furthermore, arecoline stimulates testicular Leydig cells to synthesize excessive testosterone. Arecoline has a deleterious effect on the female reproductive system, particularly impacting egg cells and contributing to premature births and poor birth outcomes during pregnancy. In animal embryo models, arecoline induces embryotoxicity, thereby impairing embryo development. Furthermore, in both urinary systems, arecoline can trigger the onset of chronic kidney disease (CKD) and exacerbate bladder cancer progression [82]. Another study indicated that arecoline induced spermatogenic damage in rats, leading to a decrease in sperm count, motility, and normal morphology [83]. As the concentration and exposure time increased, the toxic effect on sperm intensified. A decrease in sperm motility directly confirmed the reproductive toxicity of arecoline [84].

## 8. Effects on Other Organs

**Figure 3:**
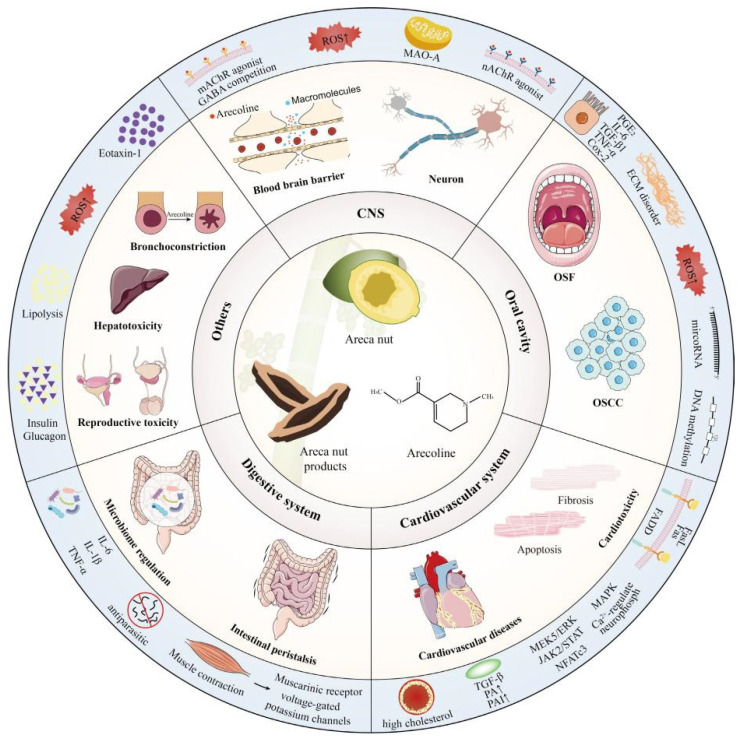
The effect mechanisms of arecoline on human organs [45].

Arecoline has different degrees of effects on the organs of the whole body. In addition to the aforementioned organs, arecoline also impacts the digestive system and immune systems, leading to intestinal inflammation, injury, and bronchial constriction. The mechanism of its influence on various organs is illustrated in Figure 3 [45]. In a study conducted by Xu et al. [24], it was observed that the consumption of arecoline led to an increase in pro-inflammatory cytokines IL-1 β and IL-6. This escalation amplified the inflammatory cascade, resulting in intestinal tissue damage and subsequent inflammation. Additionally, arecoline demonstrated hepatotoxic effects [85]. The underlying mechanism involves its influence on HIF-1 signaling, MAPK signaling, and PI3K-Akt signaling, among others, through the modulation of key targets such as ALB, CASP 3, EGFR, and MMP 9. Consequently, this modulation induces oxidative stress, triggers an inflammatory response, disrupts energy and lipid metabolism, and initiates apoptosis [86]. These actions collectively compromise the hepatocellular ultrastructure, leading to impaired liver function [39]. Experiments conducted on zebrafish embryos have demonstrated that arecoline hydrobromide induces a range of developmental abnormalities. These include delayed embryonic development, edema in the heart and yolk sacs, reduced juvenile body size, and distortion of the spinal column and tail. Additionally, the treated embryos exhibited motor retardation, decreased ocular melanin precipitation, and a concentration-dependent reduction in hatching rate [87]. Furthermore, a multitude of studies have demonstrated that maternal consumption of the areca nut during pregnancy can induce toxicity and teratogenicity in the human embryo. Yang et al. [88] conducted a study on 1264 local women who had recently undergone childbirth in 10 hospitals located in the southern and eastern regions of Taiwan Province, China. The research found that pregnancy resulted in an average reduction of 89.54 g in birth weight and 0.43 cm in length, with a significant decrease observed in male neonatal births. The study confirmed that the consumption of the areca nut, specifically its component arecoline, exposes the developing embryo to toxic and adverse effects, thereby indicating that arecoline possesses a certain level of embryonic toxicity [88]. However, research has demonstrated that arecoline can function as an antioxidant and ameliorate osteoporosis [8], Furthermore, its organic intermediate, arecoline hydrobromate, has been shown to alleviate symptoms of rheumatoid arthritis and collagen-induced arthritis [89]. Arecoline has been observed to enhance digestive processes and ameliorate gastrointestinal inflammation [12]. The upregulation of M3 mRNA levels and the increase in digestive enzyme activity have been demonstrated to enhance gastrointestinal smooth muscle tone and intestinal peristalsis, thereby facilitating digestion and absorption [90]. In rabbit-isolated small intestinal models, these effects can be observed in the promotion of smooth muscle contraction amplitude, which, in turn, protects the gastric mucosa and enhances gastric function [91].

## 9. Conclusions and Prospects

Arecoline, the primary alkaloid component in the areca nut, has garnered significant interest due to its diverse physiological effects. Based on the published scientific literature, arecoline demonstrates several favorable pharmacological effects, such as potential anti-anxiety properties at low doses, enhancement of learning and memory, promotion of gastrointestinal peristalsis, and potential benefits for osteoporosis, Parkinson’s disease, and Alzheimer’s disease. However, it also carries certain health risks, including possible harmful effects on multiple organ systems, such as the oral cavity, nervous system, cardiovascular system, and reproductive system. The bidirectional nature of arecoline’s pharmacological properties may mainly be attributed to the specific structure of the arecoline monomer and its dose effect. Further research is crucial for a comprehensive understanding of its pharmacological mechanisms and toxicological implications in order to harness the potential benefits of arecoline’s pharmacological properties. Additionally, studies focusing on reducing alkaloid toxicity are imperative for more effective and responsible drug development while safeguarding human health.

## Figures and Tables

**Table 1:** Effects of arecoline on various organs.

Num.	Action Object	Experimental Model	Experimental Result	Arecoline Dose	Reference
1	Oral system	Human umbilical vein endothelial cell	Induced endothelial mesenchymal transformation	5–20 μg/mL	[28]
2	Oral system	EAhy926 endothelial cells	Promoted oral submucosal fibrosis	>0.4 mM	[29]
3	Cerebral nerve	Sprague–Dawley rat/mouse	Induced neuronal cell death	50–200 μM	[24]
4	Cerebral nerve	Mouse	Helped regulate lipid metabolism and reduce depressive behaviors	6 mg/kg	[24]
5	Cerebral nerve	Adolescent and adult male C57BL/6J mice	Addiction	Adolescent mice: 0.03 mg/kg dose; Adult mice: 0.1 mg/kg dose	[30]
6	Cerebral nerve	Adult zebrafish	Anti-anxiety behavior	10 mg/L	[31]
7	Cerebral nerve	Zebrafish larvae	Induced hyperactivity	0.1 ppm	[32]
8	Cerebral nerve	Mouse	Enhanced learning and memory	500 mg/kg of wet *Areca catechu* extract	[33]
9	Heart and circulatory system	SD mouse	It can protect against myocardial damage caused by acute alcoholism	5 mg/(kg.d)	[34]
10	Heart and circulatory system	Sprague–Dawley mouse	It has been demonstrated that the induction of apoptosis in the heart can be achieved through the stimulation of death receptors and mitochondrial-dependent apoptosis pathways	Low dose: 5 mg/kg/day and high dose: 50 mg/kg/day	[35]
11	Heart and circulatory system	Male BALB/c mice	Promoted defecation function, enhanced gastrointestinal peristalsis	30 mg/kg	[36]
12	Heart and circulatory system	C57BL/6 mice	Caused intestinal inflammation, intestinal damage, and changes in gut microbes	6 mg/kg and 30 mg/kg	[24]
13	Embryo	Zebrafish embryos	Poor embryo survival, stunted growth	0.001–0.04%	[33,36,37,38]
14	Embryo	Pregnant mouse	In early pregnant mice, a reduction in the number of implanted embryos was observed, which resulted in the inhibition of trophoblast growth and expansion of the blastula	6.11 mg/kg/d	[39]
15	Hepatic system	Rat hepatocytes	Liver fibrosis and hepatocellular carcinoma	0.5 mM	[40]

## Data Availability

No new data were created or analyzed in this study. Data sharing is not applicable to this article.
